# Preliminary perceived intervention changes and engagement in an evidence-based program targeted at behavioral inhibition during early childhood, delivered in-person and online

**DOI:** 10.3389/fpsyg.2023.1187255

**Published:** 2023-05-26

**Authors:** Maryse Guedes, Rita Maia, Inês Matos, Marta Antunes, Teresa Rolão, Andrea Chronis-Tuscano, Kenneth H. Rubin, Manuela Veríssimo, António J. Santos

**Affiliations:** ^1^William James Center for Research, ISPA – Instituto Universitário, Lisbon, Portugal; ^2^Department of Psychology, University of Maryland, College Park, MD, United States; ^3^Department of Human Development and Quantitative Methodology, University of Maryland, College Park, MD, United States

**Keywords:** intervention program, in-person, internet-delivery, early childhood, behavioral inhibition, parenting practices, social and emotional learning (SEL) skills

## Abstract

**Introduction:**

Behavioral inhibition during early childhood is one of the strongest risk factors for the development of later anxiety disorders. Recently developed in-person interventions that target both young children who are highly inhibited and their parents (e.g., the *Turtle Program*), have decreased children's anxiety and have increased social participation in the peer group. However, researchers have yet to examine the effects of intervention mode of delivery. In the present study, we compared the pre-to post-intervention changes in child and parenting functioning of families participating in the Turtle Program, delivered in-person and online with those changes made in families allocated to a waiting-list condition; compared session attendance, homework completion and satisfaction with the intervention outcomes of families involved in the Turtle Program, delivered in-person and online; and explored the predictive role of parenting and child factors in session attendance, homework completion and satisfaction with the outcomes of families involved in the Turtle Program, depending on the mode of delivery (in-person vs. online).

**Method:**

Fifty-seven parents of highly inhibited preschoolers (3–5 years), with no diagnosis of selective mutism or developmental disorders, who were randomly allocated to waiting-list (*n* = 20), *Turtle Program* delivered in-person (*n* = 17) and online (*n* = 20) conditions completed the Portuguese versions of the *Behavioral Inhibition Questionnaire*, the *Preschool Anxiety Scale*, the *Social Behavior and Competence Scale*, the *Modified Child-Rearing Practices Questionnaire* at pre- and post-intervention assessment. Parents also completed the *Preschool Shyness Study Satisfaction Survey* at post-intervention assessment.

**Results:**

Independent of intervention mode of delivery, generalized equation estimates revealed a reduction in children's total anxiety symptoms and an improvement in parental nurturing behaviors. Child anxiety and social competence at pre-assessment were the most prominent predictors of session attendance and satisfaction with post-intervention child and parenting outcomes.

**Discussion:**

Overall, this study showed that parents in both intervention conditions perceived comparable positive changes in child functioning from pre- to post-intervention assessment and similar levels of session attendance, homework completion, and satisfaction. Significantly, however, perceived satisfaction with post-intervention child and parenting outcomes was higher, when children were reported to display higher SEL skills at baseline, independent of the intervention mode of delivery.

## 1. Introduction

Within a developmental–transactional framework, high and stable behavioral inhibition (BI) is a temperament-based wariness to the exposure to novel persons, situations, and activities (Fox et al., 2005). BI has been shown to be a precursor of social reticence with unfamiliar peers and self-imposed isolation in the peer group (anxious withdrawal, AW) at preschool (Rubin et al., 2009; Rubin and Chronis-Tuscano, 2021). The developmental cascade model from BI to social reticence and AW places children at increased risk of experiencing not only later social anxiety (Sandstrom et al., 2020) but also peer exclusion, rejection, and victimization (Rubin et al., 2018).

Developmental–transactional theory and research converge with the central tenets of developmental psychopathology, showing that not all highly inhibited preschoolers experience adverse developmental pathways (Chronis-Tuscano et al., 2009). Research to date has focused on the modifiable factors that can explain differential risk and resilience among inhibited preschoolers and that need to be targeted in early intervention programs (Danko et al., 2018). In line with the developmental–transactional framework (Rubin et al., 2009), caregiving behaviors are considered one of the main modifiable factors that can either buffer or strengthen the associations between BI and later social reticence, AW, and social anxiety (Ryan and Ollendick, 2018; Fox et al., 2023). More specifically, researchers have shown that the associations between BI and later social reticence, AW, and anxiety are strengthened by overprotective, highly controlling parenting (Rubin et al., 2002; Hane et al., 2008; Hastings et al., 2008; Lewis-Morrarty et al., 2012). In fact, this type of caregiving behavior may negatively impact the development of children's emotion-regulation skills (Fox et al., 2023) that are associated with social engagement with peers (Smith et al., 2019). Refraining from social engagement with peers may hinder children's opportunities to acquire age-appropriate social and socio-cognitive skills and to establish positive peer interactions, placing children at increased risk of adverse developmental pathways (Rubin et al., 2018).

Generated knowledge on the transactional paths among parent, child, and peer behaviors has sustained the development of evidence-based intervention programs targeting preschool children who are behaviorally inhibited (Danko et al., 2018; Rubin and Chronis-Tuscano, 2021) to enhance their social and emotional learning (SEL) skills. These abilities to understand the emotions of the self and others, regulate emotion, attention, and behavior, make good decisions regarding social problems, express healthy emotions, and engage in a range of prosocial behaviors (Denham and Brown, 2010) have been promoted through two main traditions of interventions among inhibited preschoolers (Ooi et al., 2022). The first tradition encompasses parent education programs, namely, the *Cool Little Kids* (Rapee et al., 2010), that focus on reducing overprotective and highly controlling parenting behaviors to promote children's social-approach behaviors. The second tradition includes interventions working directly with children, such as the *Social Skills Facilitated Play Program*, aimed at training social, socio-cognitive, and emotional skills in a peer group comprising inhibited preschoolers (Coplan et al., 2010; Coplan, 2020). More recently, interventions that combine both parent-focused and child-focused approaches have been introduced, such as the *Cool Little Kids* + *Social Skills Facilitated Play Program* (e.g., Lau et al., 2017), adaptations of the *Cool Little Kids* with increased child involvement (Doyle et al., 2021), or the *Turtle Program* (Chronis-Tuscano et al., 2015, 2022).

The 8-week *Turtle Program* comprises parallel parent and child groups with 5–6 families (Chronis-Tuscano et al., 2015). The parent group draws on the Parent–Child Interaction Therapy (PCIT, Eyberg et al., 2008) adapted for anxiety problems (Comer et al., 2018) and includes not only parent psychoeducational activities but also *in vivo* therapist coaching with each parent–child dyad (Danko et al., 2018). The child group extends the *Social Skills Facilitated Play Program* (Coplan et al., 2010) to teach children specific social, socio-cognitive, and emotion-regulation skills, scaffold their interactions with peers through free play and group activities, and promote children's gradual exposure to feared social situations (Danko et al., 2018). In a recent meta-analysis, Ooi et al. (2022) reported that existing evidence-based intervention programs targeting inhibited preschoolers were effective in reducing anxiety diagnoses, parent-reported anxiety symptoms, and parent or teacher-rated BI from pre- to post-intervention; intervention effect sizes were medium (Ooi et al., 2022) to large (Chronis-Tuscano et al., 2015) for these intervention outcomes. In the first trial of the *Turtle Program*, it was revealed that children in the intervention displayed significant improvements in observed peer–play interactions and social initiations and decreased teacher-reported anxiety and fear in school when compared with children allocated to a waiting-list condition (Barstead et al., 2018). With respect to parenting behaviors, a significant increase in parenting positive affect and sensitivity was found in the first trial of the *Turtle Program* (Chronis-Tuscano et al., 2015). The intervention effects of the *Turtle Program* for observed peer interactions, teacher-reported anxiety and fear, and parenting positive affect and sensitivity were of medium magnitude (Chronis-Tuscano et al., 2015; Barstead et al., 2018). Furthermore, a recent randomized controlled trial indicated that the multi-modal *Turtle Program* was more effective than the *Cool Little Kids* parent education program at modifying parent behaviors from pre- to post-intervention (Chronis-Tuscano et al., 2022).

Notwithstanding the evidence base on the effectiveness of interventions targeting inhibited preschoolers (Ooi et al., 2022), the success of such programs depends on parent engagement (Novick et al., 2020). However, few studies have examined the predictors of parental engagement in interventions targeting inhibited preschoolers (Novick et al., 2020; Bayer et al., 2021). Focusing on the sociodemographic predictors of parent behavioral engagement, Bayer et al. (2021) found that younger mothers, less educated fathers, and parents with lower household incomes were more likely to report low attendance in *Cool Little Kids*. This study also revealed that parents of girls and those from more advantaged neighborhoods were less prone to practice the learned skills. Novick et al. (2020) examined the child, parent, and intervention-level predictors of parent engagement in both the *Cool Little Kids* and the *Turtle Program*. These researchers considered both behavioral (e.g., session attendance and homework completion) and attitudinal (e.g., the degree to which the intervention is viewed as satisfactory) components. It was found that parents who participated in the *Turtle Program* displayed greater session attendance, lower homework completion, and comparable levels of satisfaction when compared with parents who participated in the *Cool Little Kids*. Few sociodemographic correlates were identified. Pre-intervention child anxiety predicted greater homework completion and session attendance, especially in the *Turtle Program*. However, pre-intervention parent depression predicted lower levels of satisfaction with the *Turtle Program*. In previous research, it has been shown that pre-intervention parenting behaviors, such as less parental praise or greater frequency of negative talk, were associated with behavioral components of parent engagement in PCIT interventions (e.g., Werba et al., 2006; Fernandez and Eyberg, 2009). Nevertheless, to the best of our knowledge, pre-intervention parenting behaviors and child SEL skills that may strengthen or buffer the associations between BI and adverse developmental outcomes (Rubin and Chronis-Tuscano, 2021) have not yet been explored as potential predictors of parent engagement in evidence-based interventions for inhibited preschoolers.

Beyond parent engagement, the accessibility of interventions targeting inhibited preschoolers has been limited by barriers related to their dissemination in the community (Morgan et al., 2016) and, more recently, to the COVID-19 crisis (Comer, 2021). To overcome these barriers, internet-delivery formats of extant interventions targeting inhibited preschoolers have been developed. *Cool Little Kids* has been adapted to a self-administered eight-module online format (Morgan et al., 2016). A pilot study showed that parents receiving either a clinician-supported (via telephone calls with a psychologist at two key points of the intervention) or a clinician-unsupported version of *Cool Little Kids Online* reported a decrease in child anxiety symptoms and diagnoses, as well as life interference (Morgan et al., 2016); the magnitude of the intervention effects was medium (Morgan et al., 2016). Furthermore, parents reported high satisfaction with the intervention (Morgan et al., 2016). The randomized controlled trial has provided additional empirical support for the effectiveness of *Cool Little Kids Online* in reducing child anxiety when compared to a wait-list condition (Morgan et al., 2017). Nevertheless, the magnitude of the decreases in overprotective parenting in *Cool Little Kids Online* was small (Morgan et al., 2017) and the frequency of program skills practice was found to be positively associated with intervention effects on child anxiety (Morgan et al., 2018).

More intensive parenting interventions drawn on PCIT and videoconferencing (e.g., the *iCALM Telehealth Program*) have been introduced to remotely deliver therapist-guided *coaching* to anxious preschoolers and their parents (Cooper-Vince et al., 2016; Comer et al., 2021). Researchers have provided evidence of high parental satisfaction and effectiveness of this internet-delivery format in reducing child anxiety and impairment when compared to a wait-list condition (Comer et al., 2021); pre- to post-intervention changes in child anxiety and impairment were small to medium (Comer et al., 2021). Research on PCIT supports the use of a group format for parents of preschool children (Barnett and Niec, 2018). More specifically, caregivers of inhibited preschoolers have been found to value the social support they received in the parent component of the *Turtle Program* drawn on PCIT adapted to anxiety problems (Danko et al., 2018; Guedes et al., 2021). Recent pilot studies have shown promising findings for internet-delivered group interventions with families of preschool children drawn on PCIT, cognitive-behavioral exposure, and videoconferencing (Hong et al., 2022). Furthermore, the developmental–transactional framework (Rubin et al., 2009; Rubin and Chronis-Tuscano, 2021) supports the need to include not only parent-focused (e.g., PCIT) but also child-focused intervention approaches to enhance children's SEL skills that increase and improve child social-approach behaviors and positive peer interactions.

Introducing an internet-delivered format of the *Turtle Program* may enhance the accessibility of evidence-based interventions targeting inhibited preschoolers in the community. Research comparing internet-delivered and clinic-based interventions, drawn on PCIT, has found positive engagement, satisfaction, and comparable intervention effects on child and parent outcomes for preschoolers with behavioral problems (Comer et al., 2017). Although anxiety disorders are the second leading mental health-related cause of disability-adjusted life years worldwide (Xiong et al., 2022), little is known about the preliminary outcomes and predictors of parent engagement in internet-delivered interventions targeting BI when compared with in-person delivered interventions.

The primary aims of the present study were to: (1) examine pre- to post-intervention changes in child and parenting functioning of Portuguese families participating in the culturally-tailored *Turtle Program* (Guedes et al., 2019a,b, 2021), delivered in-person or online, when compared with families from waiting-list conditions; (2) compare session attendance, homework completion, and satisfaction with the outcomes of families involved in the *Turtle Program*, delivered in-person and online; and (3) explore the predictive role of parenting and child factors in session attendance, homework completion, and satisfaction with the outcomes of families involved in the *Turtle Program*, depending on the mode of delivery (in-person vs. online). Drawing on prior research conducted on the *Turtle Program* in the USA (Chronis-Tuscano et al., 2015; Barstead et al., 2018) and on internet-delivered interventions targeting child BI and anxiety problems (Morgan et al., 2016; Comer et al., 2021), we expected that parents in both intervention groups would report a significant decrease in perceived child anxiety symptoms (especially, social anxiety), as well as a significant increase in perceived child social competence from pre- to post-intervention when compared with families from a waiting-list condition (H1); intervention effect changes for child anxiety and social competence were expected to be of medium magnitude (greater or equal to 0.50, Hedges, 1981). We also expected a significant improvement in self-reported nurturing parenting behaviors in both intervention groups when compared with the waiting-list condition (H2); intervention effects for self-reported nurturing parenting behaviors were expected to be of medium magnitude (≥0.50, Hedges, 1981).

To the best of our knowledge, only Comer et al. (2017) compared the effectiveness of internet-delivered and clinic-based interventions, drawn on PCIT, targeted at preschoolers who display behavioral problems. Prior research on internet-delivered interventions targeted at inhibited (Morgan et al., 2016, 2017) and anxious (Comer et al., 2021) preschoolers only included waiting-list control groups. Thus, the current state-of-the-art knowledge did not allow us to establish hypotheses concerning the differences in child anxiety symptoms and social competence, nurturing, and controlling parenting behaviors, depending on the intervention mode of delivery (in-person vs. online).

In line with prior research on the *Turtle Program* in the USA (Novick et al., 2020) and on internet-delivered interventions targeting child BI and anxiety problems (Morgan et al., 2016; Comer et al., 2021), we expected that parents in both intervention groups would display high session attendance, homework completion, and satisfaction with parent and child outcomes (H3). The current state-of-the-art knowledge did not allow us to establish hypotheses concerning between-group differences.

Based on prior research on parental engagement in the *Turtle Program* (Novick et al., 2020) and PCIT interventions (e.g., Werba et al., 2006; Fernandez and Eyberg, 2009), we expected that higher levels of child anxiety, higher levels of baseline nurturing parenting behaviors, and lower levels of controlling parenting behaviors would predict higher behavioral (i.e., session attendance and homework completion) engagement (H4). Due to the scarcity and inconsistency of research findings, we did not establish hypotheses concerning the child and parent-level predictors of satisfaction with the post-intervention outcomes and the moderating role of intervention mode of delivery in the associations between child and parent-level predictors and parent behavioral and attitudinal engagement.

## 2. Methods

### 2.1. Participants

The sample consisted of 57 primary caregivers (55 mothers and two fathers) of highly inhibited preschoolers who participated in the culturally tailored *Turtle Program* (Guedes et al., 2019a,b, 2021) delivered in-person and online. The inclusion criteria were as follows: (1) child age between 3.5 and 5 years; (2) a positive screening for BI; (3) the ability of parents and children to understand Portuguese, assessed during the pre-intervention assessment interview; and (4) parent consent and child assent to participate in the study. The exclusion criteria were a diagnosis of pervasive developmental disorders or selective mutism. In fact, pervasive developmental disorders encompass not only subjective feelings of fear and anxiety, physiological symptoms, and avoidance behaviors but also problems in social cognition, social skills, social motivation, language, and speech. Although it is typically included in broad anxiety disorders, selective mutism sometimes involves other developmental problems (e.g., developmental delays, language and speech difficulties, and autism spectrum problems). Effective interventions targeted at pervasive developmental disorders and selective mutism require not only focusing on anxiety reduction but also targeting other prototypical social, language, and speech difficulties (Muris and Ollendick, 2021).

Parent participants had a mean age of 37 years (*SD* = 3.79) and had, on average, 15 years of education (*SD* = 2.07). Most caregivers were married or cohabitating (*n* = 54, 93%) and were employed (*n* = 51, 88%). Most parents (*n* = 48, 83%) did not report having any emotional and/or behavioral problems. Children had a mean age of 55 months (*SD* = 11.77). Most children were girls (*n* = 31, 55%) and first-born (*n* = 41, 72%) and had siblings (*n* = 39, 68%). All participants reported that the children's developmental level was as expected for their age. Anxiety disorders were previously identified in three of the children, although these participants were not involved in medical or psychological treatment at the beginning of the *Turtle Program*.

Table 1 shows the baseline sociodemographic and clinical characteristics of parents who were randomly allocated to the *Turtle Program* delivered in-person (*n* = 17), the *Turtle Program* delivered online (*n* = 20), and the waiting list (*n* = 20). Parents from all groups were comparable in terms of parental age (*F* = 0.33, *p* = 0.718), sex (χ^2^ = 1.44, *p* = 0.486), years of education (*F* = 1.97, *p* = 0.148), marital status (χ^2^ = 0.43, *p* = 1.00), employment status (χ^2^ = 2.85, *p* = 0.352), and emotional/behavioral problems (χ^2^ = 0.36, *p* = 0.854). The proportion of parents who had boys and girls (χ^2^ = 1.83, *p* = 0.400) and first-borns (χ^2^ = 1.12, *p* = 0.655) was comparable across the groups. However, significant differences were found in terms of child age (*F* = 3.25, *p* = 0.047). *Post-hoc* comparisons with Bonferonni corrections showed that parents from the waiting list had significantly younger children than parents allocated to the *Turtle Program* delivered in-person. Furthermore, parents allocated to the *Turtle Program* delivered online were less likely to have other children than parents in the remaining two groups (χ^2^ = 8.44, *p* = 0.015). Parents reported comparable baseline total child anxiety (*F* = 0.87, *p* = 0.423), child social anxiety (*F* = 2.38, *p* = 0.102), and parenting restrictiveness (*F* = 2.26, *p* = 0.116). Nonetheless, significant differences were found in parenting nurturance (*F* = 5.15, *p* = 0.013). *Post-hoc* comparisons with Bonferonni corrections revealed that parents from the waiting-list reported significantly higher levels of baseline parenting nurturance than parents allocated to the *Turtle Program* delivered in-person.

**Table 1:** Sociodemographic and clinical characteristics of parents who participated in the Turtle Program delivered in-person and online.

	**Turtle Program delivered in-person (*n* = 17)**	**Turtle Program delivered online (*n* = 20)**	**Waiting-list condition (*n* = 20)**
	**M (DP) |** ***n*** **(%)**	**M (DP) |** ***n*** **(%)**	**M (DP) |** ***n*** **(%)**
Parental sex			
Mother	17 (100)	15 (89)	20 (100)
Father	0 (0)	2 (11)	0 (100)
Parental age (years)	37.07 (2.53)	36.89 (3.80)	37.80 (4.76)
Parental marital status			
Married/cohabitating	16 (94)	17 (89)	19 (95)
Other	1 (6)	2 (11)	1 (5)
Parental education (years)	16.12 (1.41)	15.00 (2.00)	15.20 (2.40)
Parental employment	17 (100)	16 (84)	17 (85)
Parental emotional problems	3 (18)	3 (16)	5 (25)
Child age (months)	60.12 (9.72)	55.58 (12.04)	50.60 (11.34)
Child sex			
Boy	6 (35)	9 (47)	11 (55)
Girl	11 (65)	10 (53)	9 (45)
Child first born	11 (64)	16 (84)	14 (70)
Child siblings	16 (94)	9 (47)	13 (65)

### 2.2. Procedures

This study is part of a pilot research project approved by the ISPA Ethics Committee.

From October 2018 to January 2020, the *Turtle Program* delivered in-person was presented to parents by pediatricians or preschool teachers from the research group's contact network and advertised in the research project's social networks. Primary caregivers (the parent who demonstrated interest in participating in the *Turtle Program*) were contacted by the research group. During the first contact, parents were informed about the study's aims and procedures. Parents who agreed to participate signed informed consent and completed the pre-assessment, which was conducted by a trained researcher. From October 2020 to October 2021, similar procedures were used to recruit participants for the *Turtle Program* delivered online.

**Figure 1:**
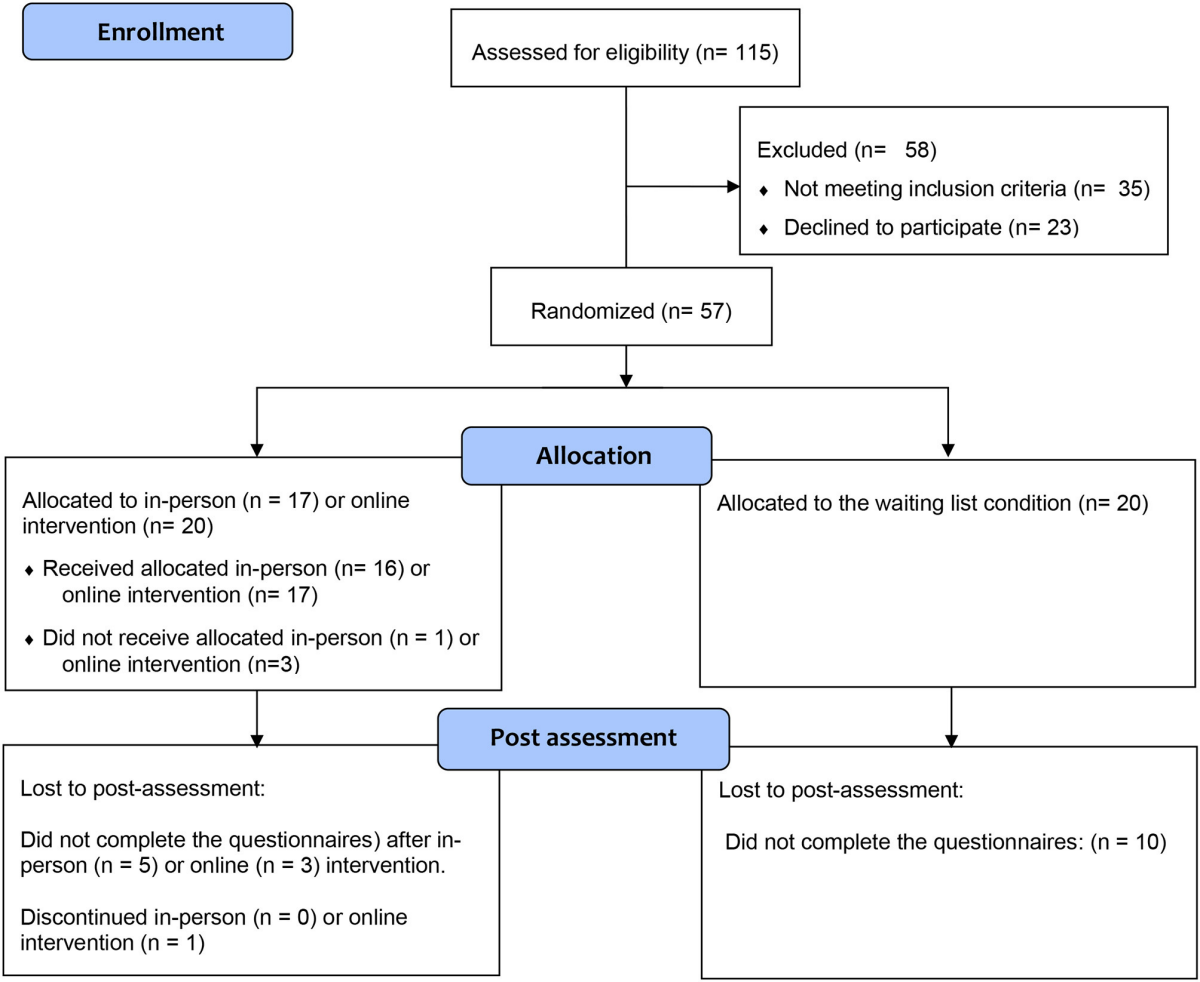
The CONSORT Flow diagram of the progress through the phases of a parallel randomized trial of the intervention (in-person and online) and waiting-list groups (that is, enrolment, intervention allocation, post-assessment). Adapted from Schulz et al. (2010).

Parents who had children who met the inclusion criteria were invited to participate in a screening interview and to complete self-report questionnaires. After the pre-intervention assessment, parents were randomly allocated to the in-person intervention, online intervention, or waiting list condition. Following the completion of the program by the intervention groups, parents were invited to complete the post-intervention assessment questionnaires. Parents from the waiting-list condition were then invited to participate in the *Turtle Program*. After the completion of the full intervention, parents from all groups were also asked to complete a satisfaction questionnaire. The post-intervention assessment was conducted by a blinded and trained researcher who did not conduct the groups with the families. The flowchart of recruitment and retention data is presented in Figure 1.

### 2.3. Intervention

#### 2.3.1. *Turtle Program* delivered in-person

This 8-weekly session intervention program (Chronis-Tuscano et al., 2015, 2022) comprised parallel parent and child groups with 5–6 families, oriented by two trained facilitators in each group.

The parent group followed the principles of the Parent–Child Interaction Therapy (PCIT, Eyberg et al., 2008) adapted for anxiety problems in children aged 2–6 years (Pincus et al., 2005; Comer et al., 2018). After a psychoeducation session on anxiety and BI, the parent group started with the first phase of the intervention (Child-Directed Interaction, CDI), during which parents learned to follow the child's lead during a 5-min special time of play. Parents were then involved in the second phase (Bravery-Directed Interaction, BDI), during which they learned the principles of gradual exposure, using hierarchies (“bravery ladders”) of feared social situations and contingent rewards for social-approach behaviors. The third phase (Parent–Child Directed Interaction, PDI) taught parents to distinguish between anxious and child-oppositional behaviors and implement effective discipline strategies (effective commands and time-out) for the latter child's behaviors. The sessions of the parent group included not only psychoeducational activities based on direct instruction, role plays, and discussion of written handouts but also *in vivo* therapist coaching of the parent and child together (Danko et al., 2018). Parents were assigned home experiences between each weekly intervention session to practice the skills learned during the parent group (identification of children's anxiety cues, special time of play, and gradual exposure to feared social situations).

The child group extended the *Social Skills Facilitated Play* Program (Coplan et al., 2010). In each session, group leaders taught briefly specific social, social problem-solving, and emotion-regulation skills, using puppets and storytelling (Chronis-Tuscano et al., 2015, 2022). Group leaders also facilitated free play and group activities, using systematic modeling and reinforcement, to scaffold children's peer interactions in an equipped playroom and enhance children's gradual exposure to feared social situations (Danko et al., 2018).

The parent and the child component of the *Turtle Program* were culturally tailored, in accordance with evidence-based recommendations in the field of developmental psychopathology (Gonzales et al., 2016) and in articulation with the research group that originally developed the intervention program. Following a multi-step approach (Gonzales et al., 2016), information on the acceptability of the intervention program was gathered, drawing on the insights of practitioners working with the targeted population (Guedes et al., 2019a,b). Based on practitioners' recommendations, minor culturally tailored modifications were introduced with the agreement of the research group who originally developed the intervention program, preliminary tested, and refined, considering parents' perspectives on the acceptability of the intervention program (Guedes et al., 2021).

In the parent component of the *Turtle Program*, intervention sessions were extended (120 min instead of 90 min) to provide more time for group discussion. Minor modifications were only introduced in psychoeducational contents and activities. More specifically, the therapists placed a greater emphasis on the evolutionary roots of BI, non-verbal communication during the CDI phase, and non-material rewards during the BDI phase. Additionally, the intervention contents were conveyed in a culturally sensitive manner, using visual support (e.g., PowerPoint, videos) and more concrete examples (e.g., challenging situations in parent–child interactions and bravery ladders). No modifications were introduced in coaching activities. Homework was renamed as home experiences. Although homework written recordings were recommended for parents' self-reflection, a greater emphasis was placed on the experiential benefits and on the relevance of sharing experiences with the group.

In the child component of the *Turtle Program*, minor modifications were introduced in the way activities were presented to children. More specifically, culturally relevant games were introduced and some intervention activities (e.g., graduation party) were renamed (e.g., bravery party).

Table 2 summarizes the structure and the contents of the eight weekly parent and child groups of the *Turtle Program*, delivered in-person.

**Table 2:** Structure and contents of the Turtle Program delivered in-person and online.

	***Turtle Program*** **delivered in-person**			***Turtle Program*** **delivered online**		
	**Parent group sessions**	**Child group sessions**	**Therapist live coaching with parents and children**	**Parent group sessions**	**Child videos and home activities with parents**	**Therapist live coaching with parents and children**
1	*Psychoeducation* on BI and anxiety	Learning to introduce yourself	Separation and pick-up	*Psychoeducation* on BI and anxiety	•Video: Expressing emotions •Home activities: puppet modeling, reading stories or watching animation films while promoting emotion knowledge	–
2	*Child-Directed Interaction teach (CDI)*	•Making eye contact •Relaxation (balloon breathing)	Separation and pick-up	*Child-Directed Interaction teach (CDI)*	•Video: Relaxation (balloon breathing) •Home activities: puppet modeling, balloon breathing in daily anxious situations	–
3	*Child-Directed Interaction (CDI) coach* during which the other parent group members observe each parent-child dyad being coached via a TV monitor.	Communicating to keep friends	Individual coach with each parent-child dyad through a bug-in-ear	*Child-Directed Interaction (CDI) coach*, during which other parent group members problem-solve special time and discuss special time videos	•Video: Learning to introduce yourself and making eye contact •Home activities: puppet modeling, practice social initiation during parent-child play	Individual coach with each parent-child dyad through bug-in-ear in a Zoom simultaneous room
4	*Bravery-Directed Interaction (BDI) teach*	Facing your fears	Separation and pick-up	*Bravery-Directed Interaction (BDI) teach*	•Storytelling: Facing your fears •Home activities: storytelling, coloring bravery ladders	–
5	*Bravery-Directed Interaction (BDI) coach 1* during which other parent group members prepare and problem-solve exposure practice.	Expressing emotions	Individual coach on an in-session bravery challenge with each parent-child dyad through a bug-in-ear	*Bravery-Directed Interaction (BDI) coach 1* during which other parent group members prepare and problem-solve exposure practice.	•Video: Communicating to keep friends •Home activities: puppet modeling, promoting sharing of interests and positive things in parent-child interactions	Individual coach on an in-session bravery challenge with each parent-child dyad through a bug-in-ear in a Zoom simultaneous room
6	*Bravery-Directed Interaction (BDI) coach II* during which other parent group members prepare and problem-solve exposure practice.	•Dealing with disappointment •“Show and tell”, observed by parents via a TV monitor	Individual coach on the preparation for the show-and-tell activity with each parent-child dyad through a bug-in-ear	*Bravery-Directed Interaction (BDI) coach II* during which other parent group members prepare and problem-solve exposure practice.	•Video: Dealing with disappointment •Session group activity: “Show and tell” with parents and children. •Home activities: puppet modeling, dealing with refusal to play from family members	Individual coach on the preparation for the show-and-tell activity with each parent-child dyad through a bug-in-ear in a Zoom simultaneous room
7	*Parent-Directed Interaction (PDI) teach*	•Working together •Scavenger hunt	Separation and pick-up	*Parent-Directed Interaction (PDI) teach*	•Video: Working together •Home activities: puppet modeling, promoting negotiation skills during play with family members	–
8	*Parent-Directed Interaction (PDI) review and planning of future*	•Review •Scavenger hunt	Graduation party involving scavenger hunt with parents, graduation ceremony and snack time	*Parent-Directed Interaction (PDI) review and planning of future*	•Video: Review •Scavenger hunt	Graduation party with scavenger hunt involving all parents and children

Partially adapted from Danko et al. (2018).

#### 2.3.2. *Turtle Program* delivered online

This 8-weekly session intervention was drawn from the culturally tailored *Turtle Program* (Chronis-Tuscano et al., 2015, 2022), delivered in-person (Guedes et al., 2019a,b, 2021) and adapted to real-time internet-delivery, by the research team, from March to October 2020.

This intervention consists of a parent group with 5–6 families, oriented by two trained facilitators on Zoom. Similar to the *Turtle Program* delivered in-person (Danko et al., 2018), the parent group follows the principles of PCIT (Eyberg et al., 2008) adapted for anxiety problems 0Comer et al., 2018). The psychoeducational activities and contents (see Table 2) are comparable to those of the culturally tailored *Turtle Program* delivered in-person (Danko et al., 2018; Guedes et al., 2019b, 2021). *In vivo* therapist coaching of the parent and the child together was adapted according to the guidelines for the internet-based delivery of the Parent–Child Interaction Therapy (Comer et al., 2015), and the CDI coaching session was delivered with each parent–child dyad individually. As in the *Turtle Program* delivered in-person, parents were assigned home exercises between each intervention session to promote the practice of the learned skills during the parent group.

Due to the children's young age, no concurrent child group was implemented in the *Turtle Program* delivered online. However, the psychoeducational contents, drawn on the *Social Skills Facilitated Play Program* (Coplan et al., 2010), targeted at children in the *Turtle Program* delivered in-person were presented in short animation videos to both parents and children at the end of each parent group session. Parents were assigned homework experiences to practice children's social, social problem-solving, and emotion-regulation skills, using puppet modeling, storytelling, and/or scaffolding in daily parent–child interactions (see Table 2). The children's group activities of the *Turtle Program* that were delivered in-person (such as show and tell and scavenger hunt) were adapted to internet-based delivery and introduced at the end of the parent group sessions to scaffold peer interaction and enhance the child's gradual exposure to feared social situations.

Families were provided detailed written information about the access to the Zoom platform, the protection measures to be implemented during the intervention sessions, and the access to the intervention materials (e.g., parent activities manual and short animation videos for children) between each intervention session. Facilitators were available before the beginning of the intervention program and 10–15 min before each of the intervention sessions to provide individual support to the families who experienced difficulties in acceding to the Zoom platform. Support was also available between each intervention session when families identified difficulties in accessing the intervention materials (e.g., parent intervention manual and short animation videos for children).

### 2.4. Instruments

During the pre-intervention assessment, the following instruments were used:

#### 2.4.1. Sociodemographic and clinical form

Parents provided information on their child (age, sex, birth order, and number of siblings) and own (age, education, and employment status) sociodemographic data. With respect to clinical data, parents were asked to report if they and/or their child were experiencing any developmental, emotional, and/or behavioral problems. If they responded affirmatively, parents reported the type of developmental, emotional, and/or behavioral problem that they and/or their child were experiencing and whether they were receiving any intervention for the reported problems.

#### 2.4.2. Selective mutism and additional childhood disorders supplementary modules—Anxiety diagnostic interview schedule for DSM-IV—Parent version

The selective mutism and additional childhood disorders supplementary modules of the ADIS-IV-P (Albano and Silverman, 1996; Russo et al., 2011) were used to conduct the screening evaluation of the exclusion criteria (i.e., diagnosis of developmental disorders or selective mutism) in the present study. The ADIS-IV-P is one of the most studied clinical interviews to assess children's anxiety disorders and other associated disorders (Silverman and Ollendick, 2005). This clinical interview has shown strong reliability in prior research with preschool children (Kennedy et al., 2009). The selective mutism module of the ADIS-IV-P includes eight yes/no questions assessing diagnostic criteria related to the child's persistent inability to speak at school (e.g., “does the child refuse to speak at school?”) and in other social situations (e.g., “does the child refuse to answer friends and other people who ask questions?”), the child's ability to speak at home (e.g., “does the child talk when he/she is at home with the rest of the family?”), the interference of the child's behavior at school (e.g., “has the school became [SIC] difficult because of his/her not talking?”) and in the family (e.g., “do you get upset because the child won't speak to other people), and the length of the reported difficulties (e.g., “has this [SIC] going on for longer than the first month of school?”). The additional childhood disorders module focusing on pervasive developmental disorders consisted of seven yes/no questions assessing diagnostic criteria related to child social interaction (e.g., “does your child has [SIC] difficulties in dealing with social interaction? For example, does he/she seem awkward in social interactions, fail to respond to others, or seem uninterested in socializing?”), communication (e.g., “does he/she has [SIC] difficulties in communicating with others? For example, does he/she delayed in his/her speech abilities [SIC], or does he/she have difficulty in initiating or following conversations?”) and ritualistic behaviors (e.g., “is your child overly preoccupied with repeating things, such as certain body movements, routines, or rituals?”), and their interference in four relevant areas of child life (school, friendships, family life, sleep, eating, and concentration).

During the pre- and post-intervention assessment, parents completed the following questionnaires:

#### 2.4.3. Behavioral inhibition questionnaire

The BIQ (Bishop et al., 2003; Fernandes et al., 2017) is one of the best-documented parent rating scales to measure children's inhibited behaviors during the preschool years (Kim et al., 2011). This rating scale corresponds with traditional laboratory observational methods for assessing BI and has been widely implemented as a stand-alone method of BI assessment (Mernick et al., 2018). The BIQ consists of 30 items that assess parent perceptions of the child's BI, considering six contexts that reflect three domains: Social Novelty (14 items), which refers to the child's inhibited behaviors toward unfamiliar adults, unfamiliar peers, and performance situations in front of others; Situational Novelty (12 items), which refers to the child's inhibited behaviors during separation and at preschool and unfamiliar situations; and Physical Activities (four items), which refers to the child's inhibited behaviors when there is a minor possible risk of injury. For each item, parents were asked to report how frequently their children displayed inhibited behaviors, using a Likert scale ranging from 1 (*Almost Never*) to 7 (*Almost Always*). Higher total scores in the BIQ indicated higher levels of child BI. Children whose mothers reported mean total scores higher than the reference mean scores plus one standard deviation (Fernandes et al., 2017) were considered eligible. Cronbach's alphas for the total score were 0.68 at the pre-intervention assessment and 0.87 at the post-intervention assessment.

#### 2.4.4. Social competence and behavior evaluation scale—Parent version (SCBE-30)

This 30-item rating scale (LaFreniere and Dumas, 1996; Fernandes et al., 2020) assessed parent perceptions about the affective quality of the relationships that children aged 30–78 months establish with peers and significant adults in context. This rating scale has been widely used in different cultures (LaFreniere et al., 2002) and provides a standardized description of affect and behavior in context, discriminating behavioral–emotional problems and social adjustment (LaFreniere and Dumas, 1996). Items were answered using a 6-point Likert scale, ranging from 1—*Never* to 6—*Always*. The SCBE-30 consists of three scales with 10 items each: Anger–Aggression, referring to externalizing behaviors; Anxiety–Withdrawal, encompassing internalizing behaviors; and Social Competence, assessing prosocial behaviors. For the purposes of the present study, we only considered Social Competence. Cronbach's alphas for Social Competence were 0.65 and 0.64 at pre-intervention and post-intervention, respectively.

#### 2.4.5. Preschool anxiety scale—Parent version

The PAS (Spence et al., 2001; Almeida and Viana, 2013) is one of the only rating scales that was specifically developed for assessing anxiety symptoms among preschoolers in accordance with the DSM-IV (Orgilés et al., 2018). This 28-item rating scale assessed parent's perceptions about the frequency of anxiety symptoms among their preschool children considering five dimensions: Generalized Anxiety, Social Anxiety, Separation Anxiety, Fears of Physical Injury, and Obsessive–Compulsive Disorder. The PAS also yields a Total Anxiety score. Parents were asked to respond to each of the presented items using a 4-point Likert scale ranging from 0—*Never* to 4—*Always*. For the purposes of the present study, we only considered the Total Anxiety score and the Social Anxiety subscale. In fact, the meta-analysis of Sandstrom et al. (2020) found that BI is a risk factor for later anxiety disorders, especially social anxiety. At the pre-intervention assessment, Cronbach's alphas were 0.91 and 0.71 for Total Anxiety and Social Anxiety, respectively. At post-intervention assessment, Cronbach's alphas were 0.91 and 0.75 for Total Anxiety and Social Anxiety, respectively.

#### 2.4.6. Child-rearing practice report questionnaire

The CRPR-Q (Rickel and Biasatti, 1982; Ribeiro et al., 2021) provides a less time-consuming assessment of child-rearing practices, in terms of broader dimensions of parenting qualities. Since its development, this self-report questionnaire has been used in a wide range of studies conducted with community and clinical samples of preschool children from different cultural settings (e.g., Andersson and Sommerfelt, 2001; Woolfson and Grant, 2006). The CRPR-Q is a 40-item self-report questionnaire that assesses parental child-rearing attitudes, values, behaviors, and goals. Four items from the original 40 items were removed because their content related to sexual issues was perceived to be inappropriate in prior studies. Parents were asked to answer each of the presented statements, considering the child who was participating in the present study. For each of the presented statements, parents rated their degree of agreement over the past month using a 6-point Likert scale from 1 (*Strongly Disagree*) to 6 (*Strongly Agree*). The CRPR-Q consists of two subscales of 18 items each. Nurturance encompasses parenting practices focusing on care, affection, and sharing feelings (e.g., “I express affection by hugging, kissing, and holding my child”). Restrictiveness refers to parenting practices focusing on the control of child behaviors (e.g., “I prefer that my child not try things if there is a chance he will fail”). Item ratings pertaining to each subscale are averaged to yield a subscale score. At the pre-intervention assessment, Cronbach's alphas were 0.77 and 0.76 for Nurturance and Restrictiveness, respectively. At post-intervention assessment, Cronbach's alphas were 0.70 and 0.81 for Nurturance and Restrictiveness, respectively.

During the intervention program, facilitators recorded the primary caregiver's attendance in each session.

At post-intervention assessment, the following instruments were used:

#### 2.4.7. Engagement in homework experiences

Parents were asked to rate how much homework they completed (Novick et al., 2020) using a 7-point Likert scale ranging from 0—*None* to 6—*All*.

#### 2.4.8. Preschool shyness satisfaction study questionnaire

This questionnaire (Chronis-Tuscano et al., 2015) consisted of 4 sections that assessed: (1) the perceived appropriateness of the intervention sessions; (2) the satisfaction with post-intervention parenting outcomes; (3) the satisfaction with post-intervention child outcomes; and (4) parental overall satisfaction with the intervention program and suggestions of improvements. For the purposes of the present study, we only examined parental responses to the questions from the second and third parts of the questionnaire. With respect to satisfaction with post-intervention parenting outcomes, parents were asked to report how much the participation in the intervention changed their parenting when their child is anxious and their satisfaction as a parent using a 7-point Likert scale ranging from 0—*Not at All* to 6—*Very Much*. In the section about satisfaction with post-intervention child outcomes, parents were asked to report how satisfied they were with progress in their child's behaviors (from 0—*Not at All* to 6—*Very Satisfied*) and to rate the evolution of child difficulties after the participation in the intervention program (from 0—*Very Much Worse* to 6—*Very Much Improved*).

### 2.5. Data analysis

Data analysis was performed using IBM SPSS Statistics, version 28.0. Descriptive statistics and comparison tests (*t*-tests and chi-square tests with Fisher correction, when applicable) were computed for sample characterization and baseline comparisons of child and parenting functioning.

Due to its robustness to small sample sizes and missing values, generalized estimating equations (GEE) were used to explore the intervention effects of the *Turtle Program* delivered in-person and online in the child (total/social anxiety symptoms and social competence) and parenting (parenting nurturance and restrictiveness) functioning when compared to the waiting-list condition. The intervention effects of the *Turtle Program* delivered in-person and online, were also examined. Unstructured correlation matrices were selected for each parameter based on the lowest quasi-likelihood under the independence model criterion (QIC) value and *a priori* hypotheses. The main effects of Time, Group, and the interaction effect of Time × Group were considered. Estimated marginal means were calculated using simple and pairwise comparisons for main and interaction effects. Effect sizes were estimated using Hedges's g (Hedges, 1981) and interpreted as: 0.2 (small), 0.5 (medium), and 0.8 (large).

ANOVAs and MANOVAs were conducted to examine session attendance, homework completion, and satisfaction with parent and child outcomes, using the intervention mode of delivery (in-person *vs*. online) as a between-subjects factor. Due to its robustness with small sample sizes (Tabachnick and Fidell, 2007), Pillai's Trace criterion (*V*) was selected for MANOVAs as the multivariate test to assess the statistical significance of the main effect of intervention mode of delivery on the set of items assessing satisfaction with parent and child outcomes.

Preliminary Pearson and point-biserial correlation analyses were conducted to identify the control variables (sociodemographic variables) and pre-intervention parenting and child variables (pre-intervention parenting nurturance and parenting restrictiveness and child BI, total anxiety, social anxiety, and social competence) that were significantly correlated with the outcomes (session attendance, homework completion, satisfaction with parent and child outcomes). When a significant correlation between a pre-intervention and outcome variable was found, moderated regression analyses were conducted in accordance with the procedures recommended by Aiken and West (1991). In the first step, control variables (child and parent sociodemographic characteristics) that were significantly correlated with the outcome were introduced, when applicable. In the second step, the pre-intervention parenting or child variable (which was centered) and the moderator (intervention mode of delivery, dummy-coded as 1—in-person and 0—online) were introduced. In the last step, the interaction term was introduced.

*Post-hoc* power calculations using G^*^Power with a significance level of 0.05 and power ≥0.80 (small: *f* = 0.10; medium: *f* = 0.25; large: *f* = 0.40; Faul et al., 2007, 2009) showed that large effects could be detected.

## 3. Results

### 3.1. Pre- to post-intervention changes in the *Turtle Program* delivered in-person, in the *Turtle Program* delivered online, and in the waiting-list condition

Table 3 summarizes the descriptive statistics (mean and standard deviations) of mother-reported child total anxiety symptoms, social anxiety symptoms, social competence and mother-reported parenting nurturance and restrictiveness in the three groups.

**Table 3:** Behavioral inhibition, anxiety symptoms, social competence, parenting practices, session attendance, homework completion, and satisfaction with child and parent outcomes in the Turtle Program delivered in-person and online.

	***Turtle Program*** **delivered in-person**		***Turtle Program*** **delivered online**		**Waiting-list condition**	
	**Baseline (*****n*** = **17)**	**Post-intervention (*****n*** = **11)**	**Baseline (*****n*** = **20)**	**Post-intervention (*****n*** = **13)**	**Baseline (*****n*** = **20)**	**Post-intervention (*****n*** = **10)**
	**M (DP)**	**M (DP)**	**M (DP)**	**M (DP)**	**M (DP)**	**M (DP)**
**Child outcomes**						
Child total anxiety symptoms	43.13 (18.21)	39.38 (17.43)	49.42 (17.06)	34.00 (11.02)	43.40 (15.46)	45.62 (11.61)
Child social anxiety symptoms	15.29 (4.81)	13.61 (4.55)	15.26 (3.71)	11.18 (2.67)	12.68 (4.30)	11.75 (3.73)
Child social competence	4.30 (0.47)	4.54 (0.60)	4.29 (0.55)	4.70 (0.44)	4.29 (0.73)	4.47 (0.73)
**Parent outcomes**						
Parenting nurturance	5.23 (0.31)	5.44 (0.27)	5.36 (0.46)	5.47 (0.33)	5.52 (0.20)	5.24 (0.21)
Parenting restrictiveness	2.88 (0.57)	2.84 (0.41)	2.74 (0.59)	2.95 (0.71)	3.12 (0.55)	3.24 (0.66)

#### 3.1.1. Pre- to post-intervention changes in the Turtle Program delivered in-person when compared with a waiting-list condition

A marginally significant Time × Group effect was found for child total anxiety. Table 4 shows parameter estimates for Time × Group effects. Child anxiety decreased from pre- to post-intervention assessment in the *Turtle Program* delivered in-person but not in the waiting-list condition. A marginally significant Time effect was found for child social anxiety and social competence. Child social anxiety symptoms decreased [*B* = 2.20, *SE* = 1.22, (CI 95%: −0.19/0.46), χ^2^ = 3.24, *p* = 0.071], whereas child social competence increased [*B* = −0.42, *SE* = 0.16, (CI 95%: −0.75/−0.10), χ^2^ = 6.37, *p* = 0.012] in both groups, from pre- to post-intervention assessment.

**Table 4:** Statistically significant main time effects for caregiver-reported child behavioral inhibition, total and social anxiety symptoms, and social competence.

	**B (SE)**	**95% CI**	**χ^2^**	***p***	***g***
**Turtle Program delivered in-person vs. Waiting-List condition**					
Child total anxiety symptoms	−9.43 (4.88)	−18.99/0.13	3.73	0.053	−0.20
Parenting nurturance	0.49 (0.23)	0.02–0.96	4.30	0.038	0.69
**Turtle Program delivered online vs. Waiting-List condition**					
Child total anxiety symptoms	−13.86 (4.92)	−23.49/−4.22	7.94	0.005	−1.00
Child social anxiety symptoms	−2.37 (1.37)	−5.04/0.31	3.00	0.083	−1.18
Parenting nurturance	0.43 (0.24)	−0.05/0.91	3.03	0.082	0.25

A significant Time × Group effect was found for parenting nurturance. As shown in Table 4, parenting nurturance increased in the *Turtle Program* delivered in-person, but decreased in the waiting-list condition. No significant main and interaction effects were found for parenting restrictiveness.

#### 3.1.2. Pre- to post-intervention changes in the *Turtle Program* delivered online when compared with a waiting-list condition

A statistically significant Time × Group effect was found for child total anxiety (see Table 4). Perceived child total anxiety increased in the waiting-list condition but decreased in the *Turtle Program* delivered online. Table 4 also shows that a marginally significant Time x Group effect was found for child social anxiety. Perceived child social anxiety tended to remain stable in the waiting-list condition but tended to decrease in the *Turtle Program* delivered online. A significant main effect of Time was found for child social competence [*B* = −0.31, *SE* = 0.12, (CI 95%: −0.53/−0.08), χ^2^ = 7.25, *p* = 0.007]. Child social competence increased in both groups from pre- to post-intervention assessment.

A marginally significant Time × Group effect was found for parenting nurturance. Parenting nurturance tended to increase in the *Turtle Program* delivered online, but decreased in the waiting-list condition. No significant main or interaction effects were found for parenting restrictiveness.

#### 3.1.3. Pre- to post-intervention changes in the *Turtle Program* delivered in-person and online

A statistically significant Time effect was found for child total anxiety. Perceived child total anxiety [*B* = 11.63, *SE* = 3.25, (CI 95%: 5.27/17.99), χ^2^ = 12.83, *p* < 0.001] and social anxiety [*B* = 3.30, *SE* = 0.78, (CI 95%: 1.78/4.82), χ^2^ = 7.26, *p* = 0.007] decreased, whereas social competence [*B* = 0.31, *SE* = 0.12, (CI 95%: −0.53/−0.08), χ^2^ = 12.83, *p* < 0.001] increased in both intervention groups from pre- to post-intervention assessment. A marginally statistically significant Time effect was found for parenting nurturance [*B* = −0.14, *SE* = 0.08, (CI 95%: −0.31/0.02), χ^2^ = 2.96, *p* = 0.086], which tended to increase in both groups. No significant Time or Time × Group effects were found for parenting restrictiveness.

### 3.2. Parent engagement in the *Turtle Program* delivered in-person and online

Table 3 displays the descriptive statistics of session attendance, perceived homework completion, and satisfaction with parenting and child outcomes of parents who provided reports on their satisfaction with the participation in the *Turtle Program* delivered in-person (*n* = 13) and online (*n* = 20). Both groups displayed comparable sociodemographic and clinical characteristics, as well as baseline child anxiety, social anxiety, social competence, and parent-reported nurturance and restrictiveness.

ANOVAs revealed that parents who participated in the *Turtle Program* delivered in-person and online reported having participated in a comparable number of sessions (*F* = 1.02, *p* = 0.319, $\eta_{\text{p}}^{2}$ = 0.031) and home skills exercises (*F* = 1.53, *p* = 0.227, $\eta_{\text{p}}^{2}$ = 0.050).

MANOVAs indicated that parents who participated in the *Turtle Program* delivered in-person and online did not report statistically significant differences in satisfaction with parenting outcomes (i.e., changes in parenting behavior and satisfaction), *V* = 0.09, *F* = 1.46, *p* = 0.251, $\eta_{\text{p}}^{2}$ = 0.094. With respect to satisfaction with child outcomes (i.e., satisfaction with child progress and perceived improvement in child difficulties), no statistically significant differences were identified between parents who participated in the *Turtle Program* delivered in-person and online, *V* = 0.06, *F* = 0.88, *p* = 0.423, $\eta_{\text{p}}^{2}$ = 0.060.

### 3.3. The predictive role of pre-intervention parenting and child factors in session attendance, homework completion, and satisfaction with parent and child post-intervention outcomes

Table 5 displays the Pearson and point-biserial correlations between the control, study, and outcome variables. Session attendance was negatively associated with pre-intervention child social anxiety and positively associated with having a first-born child and pre-intervention parenting nurturance. No significant correlations with the pre-intervention parenting or child variables and the outcomes were found. Pre-intervention child social competence was positively correlated with changes in parenting satisfaction and satisfaction with child progress. Pre-intervention child total anxiety symptoms and parenting restrictiveness were negatively correlated with perceived improvement in child difficulties.

**Table 5:** Pearson and point-biserial correlations between the control (sociodemographic and clinical), study (baseline child and parenting functioning), and outcome variables (satisfaction with post-intervention parenting and child outcomes).

	**In-person (*n* = 13)**	**Online (*n* = 20)**	**Pearson and point-biserial correlations**														
	**M (SD)**	**M (SD)**	**1**	**2**	**3**	**4**	**5**	**6**	**7**	**8**	**9**	**10**	**11**	**12**	**13**	**14**	**15**
Session attendance	6.79 (1.87)	6.09 (1.89)	−0.06	−0.13	0.32	−0.19	−0.05	0.12	−0.23	−0.24	0.37^*^	−0.09	0.14	−0.25	−0.36^*^	0.49^*^	−0.02
Homework completion	4.28 (0.77)	4.79 (0.94)	−0.08	0.32	0.13	−0.28	−0.02	−0.27	0.01	−0.07	0.47^*^	−0.02	0.23	−0.13	−0.12	0.17	0.26
Changes in parenting behavior	4.31 (1.18)	4.00 (1.02)	0.02	−0.04	−0.31	−0.27	0.04	0.13	0.03	0.11	−0.08	0.03	0.18	0.04	0.04	0.05	0.06
Changes in parenting satisfaction	4.00 (1.22)	3.06 (1.66)	−0.07	−0.14	−0.30	−0.24	−0.05	−0.03	0.08	−0.07	0.01	0.17	0.49^*^	−0.16	−0.15	0.14	−0.00
Satisfaction with child progress	4.85 (0.80)	4.78 (1.35)	−0.03	−0.12	−0.19	−0.24	0.04	−0.01	0.01	−0.07	0.11	−0.07	0.41^*^	−0.26	0.08	−0.15	−0.06
Improvement in child difficulties	4.77 (0.73)	4.44 (0.78)	−0.10	−0.02	−0.10	0.12	−0.04	−0.26	−0.13	−0.35	−0.20	0.12	0.22	−0.60^**^	−0.19	0.07	−0.43^*^

1, Parental sex (dummy coded as: 1-mother, 0 – father); 2, Parental age; 3, Parental marital status (dummy coded as: 1, married/cohabitating, 0, other); 4, Parental education; 5, Parental employment (dummy coded as: 1, employed, 0, unemployed); 6, Parental emotional problems (dummy-coded as: 1, yes, and 0, no); 7, Child age; 8, Child sex (dummy coded as 1, boy, 0, girl); 9, Child first-born (dummy coded as 1, yes, 0, no); 10, Child siblings (dummy-coded as 1-yes, 0, no); 11, Child pre-intervention social competence; 12, Child pre-intervention Total Anxiety; 13, Child pre-intervention Social Anxiety; 14, Parenting nurturance; 15, Parenting restrictiveness.

* *p* < 0.05,

** *p* < 0.01.

Table 6 shows that parents who had first-born children perceived their children as less socially anxious and reported higher levels of parenting nurturance at pre-intervention assessment and attended a higher number of sessions. No moderation effect of the intervention mode of delivery was found.

**Table 6:** Predictive role of parenting and child factors for session attendance, homework completion, and satisfaction with post-intervention parenting and child outcomes, depending on the intervention mode of delivery.

	**β**	**Δ*F***	**Δ*R*^2^**
	**Session attendance**		
**Step 1. Covariates**		4.94^*^	0.13
First-born^a^	0.36^*^		
**Step 2. Main effects**		4.15^*^	0.19
Pre-intervention child social anxiety	−0.34^*^		
Intervention mode of delivery	0.26		
**Step 3. Interaction effect**		0.01	0.00
**Step 1. Covariates**			
First-born^a^	0.29	2.82	0.09
**Step 2. Main effects**			
Pre-intervention parenting nurturance	0.49^**^	5.61^**^	0.26
Intervention mode of delivery	0.08		
**Step 3. Interaction effect**		2.59	0.05
	**Changes in parenting satisfaction**		
**Step 1. Main effects**			
Pre-intervention social competence	0.49^*^	3.72^*^	0.24
Intervention mode of delivery	−0.03		
**Step 2. Interaction effect**		0.68	0.02
	**Satisfaction with child progress**		
**Step 1. Main effects**			
Pre-intervention social competence	0.47^*^	3.41^*^	0.22
Intervention mode of delivery	−0.25		
**Step 2. Interaction effect**		1.25	0.04
	**Improvement in child difficulties**		
**Step 1. Main effects**			
Pre-intervention child total anxiety	−0.59^***^	8.03^**^	0.37
Intervention mode of delivery	0.06		
**Step 2. Interaction effect**		2.26	0.05
	**Improvement in child difficulties**		
**Step 1. Main effects**		2.99^+^	0.18
Pre-intervention parenting restrictiveness	−0.42		
Intervention mode of delivery	0.02		
**Step 2. Interaction effect**		2.03	0.06

Standardized coefficients for the interaction effect step are not presented because the inclusion of this step did not improve the percentage of explained variance.

a Dummy-coded as: 1, yes, 0, no.

** *p* < 0.01.

* *p* < 0.05.

+ *p* < 0.10.

Additionally, parents who described their children as more socially competent at pre-intervention assessment reported greater changes in parenting satisfaction and higher levels of satisfaction with child progress post-intervention. The intervention mode of delivery did not moderate the associations between the study and outcome variables.

Finally, parents who perceived their children as less anxious at pre-intervention assessment reported greater improvements in child difficulties. No moderation effect of the intervention mode of delivery was found.

## 4. Discussion

To the best of our knowledge, this is the first study to examine perceived pre- to post-intervention changes in child and parenting functioning and the engagement of families involved in the culturally tailored *Turtle Program* delivered in-person and online in a European country and to explore the predictive role of child and parenting factors for caregivers' engagement, depending on the intervention mode of delivery.

Our findings are partially consistent with our first research hypothesis (H1). Independent of the intervention mode of delivery, our findings show that participation in the *Turtle Program* seems to be associated with a reduction in total anxiety and social anxiety symptoms from pre- to post-intervention. The higher magnitude of the reduction in parent-reported total child anxiety symptoms in both intervention conditions when compared with a waiting-list condition is consistent with the findings of the pilot randomized controlled trial of the *Turtle Program* (Chronis-Tuscano et al., 2015) delivered in-person that was conducted in the USA. These findings are also in line with the main conclusions of a meta-analysis conducted by Ooi et al. (2022) concerning the effectiveness of existing in-person and online evidence-based interventions targeting inhibited preschoolers in decreasing parent-reported total anxiety symptoms.

Contrary to our hypothesis (H1), the magnitude of the decrease in parents' reports of social anxiety symptoms was only higher than in the waiting-list condition among caregivers who participated in the *Turtle Program* delivered online. This finding is inconsistent with the results of the pilot randomized controlled trial of the *Turtle Program* (Chronis-Tuscano et al., 2015) delivered in-person in the USA. The findings reported herein need to be interpreted with caution due to the small sample sizes and attrition in the completion of post-intervention measures. Nonetheless, the population-level *Cool Little Kids* dissemination trial (Bayer et al., 2018) and some of its recent adaptations (Doyle et al., 2021), delivered in-person, have also found that the decrease in mother-reported anxiety symptoms from pre- to post-intervention assessments was comparable in the intervention and control group conditions.

Children in the waiting list condition were significantly younger than children in the *Turtle Program* delivered in-person. During the preschool years, there is generally an increase in the number of naturally occurring exposures to feared social situations (Doyle et al., 2021). According to the developmental–transactional framework (Rubin et al., 2009; Rubin and Chronis-Tuscano, 2021), it is possible that family anxiety accommodation that maintains and strengthens children's difficulties is less accentuated and generalized among caregivers of younger preschoolers. Furthermore, families were recruited not only through healthcare practitioners and preschool teachers from the contact network of the research group but also through advertisements in the social networks of the research project. Doyle et al. (2021) hypothesized that caregivers in the control groups may reflect on their children's social anxiety after pre-intervention assessments, search, and apply available psychoeducational information on child social anxiety, such as those accessible in the social networks of our research project. These factors may have diluted the intervention effects of the *Turtle Program* delivered in-person. On the other hand, the *Turtle Program* delivered online included parent-child home experiences drawn on the didactic portion of the *Social Skills Facilitated Play* (Coplan et al., 2010) to enhance children's emotion-regulation and social skills. Given that they were more directly involved in the promotion of children's emotional knowledge, expression, and regulation, it is possible that parents in the *Turtle Program* delivered online were more aware of changes in children's social anxiety symptoms, such as worries about doing something embarrassing in front of other people or fearing to meet or talk to unfamiliar people.

In this study, it was also found that parents' reports of children's social adjustment in the peer group (i.e., children's abilities to consider the perspectives of others and the demonstration of cooperation in the peer group) showed an improvement from pre- to post-intervention assessment in the *Turtle Program* delivered in-person and, to a lesser extent, in the *Turtle Program* delivered online. In the *Turtle Program* delivered in-person, group leaders facilitated free play and group activities in an equipped playroom with a group of peers with similar difficulties (Danko et al., 2018). Social play is a core developmental context during the preschool years and its quality impacts key protective factors (e.g., peer acceptance and reciprocal friendships) for healthy socioemotional outcomes (e.g., Coelho et al., 2017) among children who are behaviorally inhibited (e.g., Sette et al., 2017). In contrast, children in the *Turtle Program* delivered online mainly participated in virtual group activities (e.g., show and tell and scavenger hunt) with adults and inhibited peers. These differences between the two intervention conditions may have influenced the reported findings.

Contrary to our hypothesis (H1), improvement in perceived child social competence from pre- to post-intervention assessment was also observed in the control group. These findings are inconsistent with prior research, showing the beneficial effects of the *Turtle Program* delivered in-person (Barstead et al., 2018) and the *Social Skills Facilitated Play Program* (Coplan et al., 2010; Li et al., 2016) in children's peer interaction and prosocial behaviors when compared with a waiting-list condition. This may be explained by methodological differences between the studies. Prior studies about the *Turtle Program* delivered in-person (Barstead et al., 2018) relied on the reports of preschool teachers and trained observers in naturalistic peer play contexts. Although parents observe qualitatively different behaviors and are more familiar with children's verbal and non-verbal cues in multiple contexts, teachers observe children in daily activities with familiar peers for a significant amount of time and they develop standards of competent behaviors based on their observation of many children of similar age and their academic knowledge pertaining to child development (Fernandes et al., 2020). Given that rating scales presuppose that informants judge how a child typically behaves in comparison with others retrospectively (Fernandes et al., 2020), observational measures are frequently considered the gold standard in intervention research (Chronis-Tuscano et al., 2015; Barstead et al., 2018). Furthermore, the increase in peer play behaviors among inhibited children from waiting-list conditions has been also found in prior research (Barstead et al., 2018) and has been suggested to reflect the natural “warming up” that occurs when providing sufficient exposure over time (Rubin and Krasnor, 1980).

In line with our second research hypothesis (H2), statistically significant differences were found from pre- to post-intervention assessment in self-reported parenting nurturance in both intervention conditions when compared with the control condition. Prior research on the *Turtle Program* has been based on observational assessments of parenting behaviors during free play and structured tasks (Chronis-Tuscano et al., 2015). Although parent reports on their caregiving behaviors may be biased, our findings are consistent with prior research about the *Turtle Program* in the USA, showing a significant improvement in observed parental positive affect, sensitivity (Chronis-Tuscano et al., 2015), and positive engagement with the child during free play and structured tasks (Chronis-Tuscano et al., 2022). Furthermore, these findings are in line with the beneficial intervention effects of PCIT targeted at socially anxious children (Comer et al., 2021). Nevertheless, the magnitude of perceived changes in parental willingness to listen and share experiences with their children, and demonstrate affection, acceptance, and responsiveness toward their children's needs (Rickel and Biasatti, 1982) was greater, relative to a waiting-list, for the in-person than the online *Turtle Program*. These findings need to be interpreted with caution because pre-intervention assessment differences were identified between the in-person intervention and waiting-list conditions.

The few studies comparing clinic-based and internet-delivered PCIT indicated comparable intervention effects but were targeted at individual families with children who displayed externalizing behaviors (Comer et al., 2017). Prior research has suggested that clinic-based PCIT may provide more opportunities for therapists to build rapport with the child and to model skill use with parents, so that the parents' learning process may be lengthened (Comer et al., 2015). These additional challenges to build rapport with the child may be particularly salient with families of inhibited preschoolers who typically display increased emotional reactivity and wariness when exposed to unfamiliar adults (Fox et al., 2005). Although brief interactions with the parent–child dyads were introduced in the *Turtle Program* delivered online to counteract these potential issues (Comer et al., 2015; Cooper-Vince et al., 2016), therapist modeling is also more limited to the parent–child dyad coaching sessions than in the *Turtle Program* delivered in-person, where parents are also coached during separation and pick-up (Danko et al., 2018). This may explain why the magnitude of the differences in parenting nurturing behaviors when compared with the control group condition were lower in the *Turtle Program* delivered online.

In contrast with the second hypothesis (H2), our study did not identify significant pre- to post-intervention changes in parenting restrictiveness. Nonetheless, our findings are in line with the pilot study of Chronis-Tuscano et al. (2015) that found a lack of intervention effects in negative/intrusive control and attributed them to the characteristics of the sample (i.e., low negative control at the baseline) and the observational context. Although the internet-delivery format of the *Turtle Program* is different, the randomized controlled trial of *Cool Little Kids Online* only found small magnitude reductions in self-reported specific parenting overprotective/overinvolved behaviors that discourage autonomy in young children (Morgan et al., 2018). In our sample, parents reported parenting restrictiveness, that is, their degree of control toward children's behaviors and feelings, the establishment of narrow limits on children's behaviors, and the endorsement of strict rules, requirements, and restrictions (Rickel and Biasatti, 1982) at pre- and post-intervention assessment. Intervention changes in overprotective parenting behaviors that can increase the risk of adverse developmental pathways among inhibited preschoolers (Rubin et al., 2009; Rubin and Chronis-Tuscano, 2021) may have not been identified through parent self-reports in our study. On the other hand, parents who participate in the *Turtle Program* are taught the contributions of parenting behaviors to children's anxious behaviors and receive therapist feedback about their caregiving behaviors during child-led play and graduated exposure practice (Danko et al., 2018). Recent research on PCIT for internalizing problems has found that parents seem to become more aware of their own caregiving behaviors and display more accurate self-perceptions of parenting at post-intervention assessment (Whalen et al., 2021). In a qualitative study, parents who participated in the *Turtle Program* delivered in-person acknowledged that they became more aware of parenting behaviors that maintained and strengthened children's inhibited behaviors at post-intervention assessment (Guedes et al., 2020). This may have influenced caregivers' self-reported parenting restrictiveness at post-intervention assessment in our sample.

Consistent with the third hypothesis (H3), parents in our sample had high session attendance, reported moderate homework completion (between 65 and 80%), and were satisfied with the progress in children's behaviors, considering that children's anxious behaviors and their ability to manage them improved after the participation in the *Turtle Program*. Parent behavioral (session attendance and homework completion) and attitudinal (satisfaction with parenting and child outcomes) engagement was comparable among parents participating in the *Turtle Program* delivered in-person and online. These findings are consistent with prior research on PCIT showing that parents who participated in clinic-based and real-time internet delivery formats reported comparable session engagement and satisfaction with the intervention (Comer et al., 2017). Perceived changes in caregivers' satisfaction as a parent after the participation in the *Turtle Program* delivered in-person and online were neutral to moderate in both intervention groups. Parental satisfaction refers to parental feelings of frustration, anxiety, and motivation in the parenting role (Johnston and Mash, 1989). Caregivers' emotions and cognitions toward children's inhibited behaviors are explored during the Bravery-Directed Interaction (BDI) phase of the *Turtle Program*, but most of the intervention activities are focused on the modification of parenting behaviors for the promotion of children's independence and SEL skills to approach anxiety-inducing situations (Danko et al., 2018). Consequently, parents may have noticed less intervention changes in their parental satisfaction than in their ability to manage children's anxious behaviors, at least immediately after their participation in the *Turtle Program*.

Consistent with the study of Novick et al. (2020), few sociodemographic correlates of parent engagement were identified in our sample. Our findings only showed that having a first-born inhibited child was associated with greater parent behavioral engagement. Previous parenting experience has been found to be associated with higher levels of parenting knowledge about childrearing and child development (Bornstein et al., 2022). This may have influenced parents' engagement in the sessions and home experiences of the developmentally grounded *Turtle Program*.

In our study, baseline child anxiety and SEL skills seemed to be the most prominent predictors of parent engagement in the *Turtle Program* delivered in-person and online. More specifically, our findings show that parents who rated their child as more socially anxious at pre-intervention assessment attended a lower number of intervention sessions. These findings are inconsistent with our hypothesis (H4) and with the study of Novick et al. (2020). In fact, Novick et al. (2020) concluded that higher levels of clinician-rated impairment due to child anxiety disorders predicted greater session attendance in the *Turtle Program* delivered in-person. These divergences in the obtained findings may be associated with methodological and informant differences. In our study, parents reported domain-specific and total anxiety symptoms. In the US study, clinicians rated global impairment due to child anxiety disorders (including separation, social, specific, and generalized anxiety disorders) (Novick et al., 2020). On the other hand, the intervention features may have influenced our findings. More specifically, the *Turtle Program* delivered in-person involves *in vivo* coaching activities with each parent–child dyad and concurrent child activities in a peer group with similar difficulties (Danko et al., 2018). Although it does not include a concurrent child group, the *Turtle Program* delivered online presupposes child involvement in parent–child *coaching* and group activities in the parent sessions. This exposure to unfamiliar adults and peers and performance situations in front of others is anxiety-inducing for inhibited preschoolers (Bishop et al., 2003), especially for children who are perceived as more socially anxious (e.g., are worried about doing something embarrassing in front of other people or are afraid to meet or talk to unfamiliar people) by their parents. When inhibited children display increased socially anxious behaviors, the developmental–transactional framework (Rubin et al., 2009; Rubin and Chronis-Tuscano, 2021) acknowledges that parents often perceive them as vulnerable, accommodate their anxiety, and engage in avoidance behaviors, refraining from encouraging them to engage in developmentally relevant social opportunities (Chronis-Tuscano et al., 2018) and, possibly, in the intervention sessions.

Our findings show that parenting nurturing behaviors predicted greater session attendance independent of the intervention mode of delivery. This is in line with our hypothesis (H4) and prior research on PCIT, showing that higher levels of positive parenting behaviors (such as parental praise) are associated with increased parent behavioral engagement in PCIT interventions (Werba et al., 2006; Fernandez and Eyberg, 2009).

Parents in our sample who reported higher levels of pre-intervention child total anxiety reported lower improvements in child difficulties. Children with higher levels of total anxiety symptoms not only display increased worries and fear in social situations but also generalized worries, physical injury fears, and difficulties during parent–child separations (Spence et al., 2001). As established in the developmental–transactional framework (Rubin et al., 2009; Rubin and Chronis-Tuscano, 2021), parents of inhibited children who display difficulties in a wider range of situations and contexts may perceive them as more vulnerable and engage in more parental control and overprotection behaviors that maintain or even exacerbate children's difficulties (Hastings et al., 2019). In the present study, it was found that baseline parenting restrictiveness is negatively correlated with perceived post-intervention improvements in child difficulties. Furthermore, the modification of child's anxious behaviors in the *Turtle Program* delivered in-person and online followed the principles of cognitive-behavioral exposure, using hierarchies of anxiety-inducing situations (“bravery ladders”) and contingent social rewards for approach behaviors (Danko et al., 2018). Cognitive-behavioral exposure presupposes graduated and repeated practice across time, which requires a commitment on the part of the families beyond attending the sessions (Seligman and Ollendick, 2012). This may have been especially challenging for those families who reported that their children experience anxiety in a wide range of situations and contexts. It is possible that these families were not able to practice graduated and repeated exposure to the wide range of anxiety-inducing situations for their children, during the 8-week *Turtle Program*. This may explain why these parents perceived lower improvements in child difficulties.

With respect to child SEL skills, our findings revealed that parents who perceived that their children displayed higher levels of social competence at the pre-intervention assessment reported greater changes in post-intervention parental satisfaction and higher levels of satisfaction with child progress. Within a developmental–transactional framework (Rubin et al., 2009, 2018; Rubin and Chronis-Tuscano, 2021), children's abilities to regulate emotions, engage in a wider range of prosocial behaviors, and make good decisions about social problems have been recognized as protective factors that enhance healthy developmental pathways among inhibited preschoolers. Children's positive adaptative qualities at the pre-intervention assessment may have facilitated the practice of graduated exposure to anxiety-inducing situations within and outside the intervention sessions. When inhibited children are perceived as less vulnerable in social situations (e.g., in peer play contexts), parents may be less likely to respond to them in an overprotective, controlling, and directive manner and may be more prone to encourage children's engagement in social situations (Chronis-Tuscano et al., 2018) within and beyond the intervention sessions. Furthermore, children who display more emotionally mature and prosocial behaviors in peer play contexts may also be more likely to engage in the child didactic and group activities of the *Turtle Program* delivered in-person (Danko et al., 2018) and online. This may influence positively caregivers' satisfaction with child outcomes and changes in parenting satisfaction.

Study limitations need to be acknowledged. In the present study, the sample size at the baseline was comparable to the sample sizes observed in prior pilot randomized controlled trials conducted in clinic-based (e.g., Coplan et al., 2010; Chronis-Tuscano et al., 2015; Barstead et al., 2018) and internet-delivered interventions (e.g., Donovan and March, 2014; Comer et al., 2021) targeting inhibited and anxious preschoolers, but the attrition in the completion of post-intervention measures was higher. In line with prior research (e.g., Donovan and March, 2014; Chronis-Tuscano et al., 2015; Comer et al., 2021), we found significant and sizeable pre- to post-intervention changes in child anxiety symptoms and parenting nurturing behaviors in the intervention conditions when compared with a waiting list condition using GEE. Nevertheless, the small sample size and the attrition in the completion of post-intervention assessments may have underpowered the detection of between-group differences in child prosocial behaviors that were found in prior studies (Coplan et al., 2010; Barstead et al., 2018) and the detection of moderation effects of intervention mode of delivery in parent engagement. In fact, *post-hoc* power calculations indicated that large effects could be detected. Even if both intervention groups did not differ significantly in terms of baseline characteristics, we cannot ignore that the delivery of the *Turtle Program* in-person and online took place at different time intervals. More specifically, the *Turtle Program* was delivered online during the second, third, and fourth waves of the COVID-19 pandemic crisis. The real-time internet delivery of the *Turtle Program* allowed us to reach parents from other Portuguese regions who would not be able to attend the intervention delivered in-person conducted in the Metropolitan Lisbon area. Nevertheless, the timing during which the online *Turtle Program* was delivered and the percentage of the Portuguese population aged 16–74 years who display digital literacy skills classified as basic and beyond (Eurostat, 2022) may have influenced both perceived intervention outcomes and parental engagement. Although we assessed both positive and negative domains of child and parental functioning, our findings reflected the perspectives of parents (mostly mothers) using validated rating scales, self-report questionnaires, and specific items (e.g., satisfaction with child and parenting outcomes) developed by the research group who developed the *Turtle Program* (Novick et al., 2020). Maternal ratings about children's behaviors may be influenced by memory bias, as well as by maternal knowledge and beliefs toward the assessed behaviors (Fernandes et al., 2020). Furthermore, mothers' ability to report their own parenting behaviors may be influenced by social desirability biases, leading mothers to under-report negative behaviors while overreporting positive behaviors (Whalen et al., 2021). Although the COVID-19 crisis negatively impacted the conduction of in-person assessments, the absence of observational measures of child BI and parenting behaviors in the present study is noteworthy. Despite its limitations, this is the first preliminary study to examine pre-post intervention changes in child and parenting functioning in the *Turtle Program* delivered in-person and online when compared with a waiting-list condition. The findings of our study are encouraging that the *Turtle Program* could be delivered in-person and online in a cultural context different from the venue within which the intervention was developed (the USA). Future randomized controlled trials with diverse active control groups (e.g., in-person parent-only or child-only interventions and self-administered internet-delivery interventions) should be conducted. These trials need to include larger samples with more diverse sociodemographic characteristics, use a multi-informant (e.g., parents, teachers, and trained observers) and multi-method (e.g., observations of parenting and child social behaviors, questionnaires, and diagnostic interviews) approach, and introduce follow-up assessments to better understand the long-term effects of the intervention. This may allow for the examination of mediators or moderators of the intervention effects, depending on the intervention mode of delivery. Deepening the understanding of parent engagement and its predictors requires a more comprehensive measurement of both behavioral and attitudinal components (e.g., weekly homework completion and satisfaction), the inclusion of other parent-level factors (e.g., parental stress, mental health diagnoses, and beliefs about child inhibited behaviors) and the examination of the interaction between child and parent-level factors. This may guide the development of add-on motivational modules to enhance parent engagement and, ultimately, intervention effectiveness.

Overall, in line with a transactional–developmental framework (Rubin et al., 2009; Rubin and Chronis-Tuscano, 2021), our findings provide further evidence of the beneficial effects of early multimodal interventions targeted at BI to reduce parent-reported child anxiety symptoms and promote nurturing parenting behaviors that can place inhibited preschoolers in healthier developmental pathways. The promising beneficial effects of the therapist-guided online *Turtle Program* extend the current state-of-the-art knowledge and need to be further investigated. In fact, this type of internet-delivered intervention program targeted at inhibited preschoolers has the potential to maximize intervention cost-effectiveness and to minimize barriers to intervention adherence and persistence among families who live in areas with limited access to mental health services and/or experience attendance difficulties due to diverse factors (e.g., scheduling of the sessions, transportation issues, and siblings' childcare arrangements).

Our findings also appear to highlight the importance of a multi-domain developmental assessment before the intervention to understand children's difficulties and identify individual protective factors (namely, children's SEL skills) against unhealthy developmental outcomes. This assessment can guide the design of evidence-based motivational strategies that can enhance parent engagement in multimodal intervention programs targeted at BI delivered in-person and online. More specifically, a greater focus on psychoeducation about the parenting behaviors (e.g., parenting accommodation and avoidance of social situations) that maintain BI and on the cognitive restructuring of unrealistic expectations for immediate changes in children's behaviors may be needed for parents who perceive that their inhibited children display more anxiety symptoms and less SEL skills at the baseline.

## Data availability statement

The raw data supporting the conclusions of this article will be made available by the authors, without undue reservation.

## Ethics statement

The studies involving human participants were reviewed and approved by Comissão de ética do ISPA. Written informed consent to participate in this study was provided by the participants' legal guardian/next of kin.

## Author contributions

MG, AS, MV, AC-T, and KR contributed to conception and design of the study. MG, RM, IM, MA, and TR collected the data. MG performed the statistical analysis and wrote the first draft of the manuscript. All authors contributed to manuscript revision, read, and approved the submitted version.
